# Comparative Analysis of In Vitro Permeation and Human Pharmacokinetic Data for a Bioadhesive Vaginal Tablet as a Potential Clinical Approach for Bacterial Vaginosis

**DOI:** 10.3390/pharmaceutics18091183

**Published:** 2026-09-19

**Authors:** Monika Kłosińska, Anna Kiryluk, Bartłomiej Kubiak, Emilia Szymańska

**Affiliations:** 1 Research and Development Department, Adamed Pharma S.A., Pieńków, Mariana Adamkiewicza 6A, 05-152 Czosnów, Poland; 2 Department of Pharmaceutical Technology, Medical University of Białystok, Mickiewicza 2c, 15-222 Białystok, Poland

**Keywords:** bacterial vaginosis, bioadhesive vaginal tablet, first-in-human study, in vitro permeation test, pharmacokinetics

## Abstract

**Background/Objectives:** Reformulating oral drugs for vaginal delivery is a current therapeutic strategy for genitourinary tract infections. Bioadhesive delivery systems, by interacting with the mucosal epithelium, improve drug deposition at the application site and are promising carriers for drugs with poor bioavailability. This study compares an in vitro permeation test (IVPT) in excised human vaginal epithelium and a first-in-human (FIH) pharmacokinetic (PK) study for a bioadhesive vaginal tablet developed as a potential clinical approach for bacterial vaginosis (BV). **Methods:** A Phase 1, single-dose, open-label, crossover study in 24 healthy volunteers evaluated PK differences after ascending doses of the vaginal tablet and an oral comparator, with a 7-day wash-out between administrations. The IVPT examined drug permeation and deposition in excised human vaginal epithelium for the pure drug and drug-loaded tablet. **Results:** IVPT showed limited penetration of the antimicrobial agent across human vaginal epithelium. Tablet excipients reduced drug accumulation in the ectocervical tissue, further hindering absorption. Single vaginal administration resulted in low plasma drug levels (C_max_ 0.084–0.127 ng/mL), irrespective of dose. Mean AUC_(0__–t)_ after vaginal tablet administration did not exceed 1.560 h·ng/mL (100 mg) and was significantly lower than for the oral comparator (824.147 h·ng/mL, 100 mg). The IVPT data with human vaginal epithelium supported the FIH data, demonstrating low systemic absorption following single-dose vaginal application and potential for local drug performance. **Conclusions:** A single dose of the bioadhesive vaginal tablet appears to be a potentially safe alternative for BV treatment, with minimal systemic drug exposure, supporting further research on clinical efficacy and local pharmacodynamics of bioadhesive tablets for BV.

## 1. Introduction

Vaginal drug delivery is preferable for the local therapy of genitourinary tract infections (bacterial vaginosis, candidiasis, sexually transmitted diseases). Increasing research is focused on reformulating oral drugs for vaginal delivery, aiming for reduced side effects, and improving the activity of poorly soluble or unstable drugs [1]. Effective treatment, particularly for infectious diseases, requires adequate drug contact with the vaginal epithelium. The limited drug residence in the vaginal mucosa often leads to treatment failure, recurrent infections, and the development of antimicrobial resistance [2]. Bioadhesive delivery systems interact with the mucosal epithelium to prolong drug residence time but may also affect absorption rate and systemic availability. Understanding the strengths and limitations of vaginal bioadhesive carriers is therefore crucial for safety considerations.

A comprehensive evaluation of the pharmacokinetic (PK) profile is essential for novel vaginal formulations to elucidate drug absorption, distribution, and elimination pathways. To date, relatively limited human studies have been reported on the safety and pharmacokinetic profile after vaginal delivery [3,4,5,6,7]. Conducting human trials poses several challenges, mostly resulting from the highly variable vaginal microenvironment across individuals.

In Vitro Permeation Testing (IVPT) is a regulatory-accepted alternative to in vivo studies for predicting bioavailability and safety, helping reduce animal and human testing in the development of topical drugs. Among the available barrier models, human explants serve as a physiologically relevant IVPT approach for decision-making during early-stage development [8,9]. Over the past few years, published studies have provided insight into the implementation of IVPT in the development phase of topical delivery systems [10,11,12]. To date, only a limited number of studies have been conducted on the absorption profile through excised human vaginal epithelium [13,14]. More importantly, there is a clear gap between in vitro drug permeation and in vivo pharmacokinetics following vaginal application. To address this issue, this work reports IVPT using human excised vaginal tissue, followed by a first-in-human (FIH) study evaluating systemic absorption after a single dose of a bioadhesive vaginal tablet developed for the treatment of bacterial vaginosis (BV). The novel vaginal formulation was reformulated from an oral medicinal product registered for genitourinary tract infections. The antimicrobial component utilised in the vaginal tablet is a poorly water-soluble, broad-spectrum bacteriostatic agent with a short half-life after oral administration. The primary objective was to investigate the PK differences between various strengths (5, 25, and 100 mg, where preliminary data on the minimum inhibitory concentrations for BV pathogens observed in vitro played a key role in guiding dose selection) of the vaginal tablets and the oral comparator (100 mg), and to evaluate the impact of dose on drug absorption. The secondary objective was to assess the safety and tolerability of the investigational products.

## 2. Materials and Methods

### 2.1. Materials

Ammonium acetate, acetonitrile (ACN), and HPLC-grade dimethyl sulfoxide (DMSO) (Avantor Performance, Gliwice, Poland) were sourced. Hydroxyethylcellulose (HEC) (Fluka), isotonic saline solution (Polpharma SA, Starogard Gdański, Poland), octylphenoxypoly(ethyleneoxy)ethanol, and sodium azide (Sigma Aldrich, Hamburg, Germany) were also obtained. Simulated vaginal fluid (SVF, pH 4.5) was prepared as described in [15].

### 2.2. Study Product

Bioadhesive vaginal tablets contained 5, 25, or 100 mg of the poorly soluble drug (0.01 mg/mL in water, 20 °C), with a molecular weight of 264.2 g/mol, pKa 8.23, and logP 0.6. The bioadhesion was assessed using a TA.XT.plus Analyser (Stable Micro Systems, Godalming, UK) with a thermostated Mucoadhesion Rig at 37 °C. A tablet wetted with SVF and a 10% mucin solution were used as an in vitro mono-component mucosa model.

### 2.3. IVPT Study

IVPT was performed during the development stage to check how the drug penetrates the vaginal epithelium. The Bioethics Committee of the Medical University of Bialystok (Poland) revised and approved the study protocol for the use of human tissue under the number (No R-I-002/462/2018). Freshly excised ectocervical epithelium was obtained from the Private Clinic of Obstetrics and Gynecology in Bialystok between January and March 2019 from five healthy premenopausal women undergoing gynaecological plastic surgery. Epithelial tissue with any signs of abnormality was excluded. No information concerning the patient’s identity, except for age, was disclosed. The studies were conducted in an in-line cell system equipped with Bronaugh diffusion chambers. The IVPT model investigated the ‘isolated’ absorption profile of a pure drug and a powdered vaginal tablet suspended in SVF/HEC. For this purpose, a suspension of pure drug (5 mg) or tablet mass (whose amount corresponded to the drug dose of 5 mg) was placed on the vaginal epithelium. The tablet disintegration and drug dissolution stages were excluded as rate-limiting steps to distinguish the effect of tablet excipients solely on drug penetration. The presence of SVF/HEC ensured homogeneous distribution of drug or tablet mass on the tissue surface [16] and had no impact on epithelial transport [17,18]. IVPT parameters are presented in Table 1.

#### Sample Handling and Laboratory Assay

Immediately after the surgery, human vaginal tissue specimens were preserved in isotonic saline solution and stored at −20 °C for no longer than 60 days. Excised human vaginal epithelium, thawed at room temperature, was placed between the donor and acceptor compartments in in-line diffusion cells with the epithelial side facing the donor compartment and equilibrated in isotonic saline solution for 1 h. After the equilibration period, a proper amount of pure drug, or powdered vaginal tablet, was suspended in SVF with the addition of HEC and placed in the donor compartment immediately. The donor compartments were protected with parafilm. The acceptor medium was then recirculated beneath the vaginal tissue at a constant rate of 20 mL/h. At predetermined time intervals, the acceptor medium was collected, filtered, and analysed for drug content using reversed-phase HPLC with UV detection. The samples were replaced by the same volume of acceptor solution to ensure sink conditions. At the end of the studies, the suspension from the donor compartment was aspirated and washed with SVF and DMSO until the drug was completely removed from the tissue. The aspirate was then diluted with a mixture of DMSO/ACN/water and analysed for drug content. The tissue was homogenised, extracted with a DMSO/ACN/water mixture (30 °C), and analysed for drug accumulation by HPLC. Results were statistically assessed using the nonparametric Mann–Whitney test in Statistica 12.0 (StatSoft Poland). Quantitative variables were expressed as mean ± standard deviation (SD) by using MS Excel software. Measurements were considered significant at *p* < 0.05.

### 2.4. Clinical Study

#### 2.4.1. Study Design and Participants

**Figure 1:**
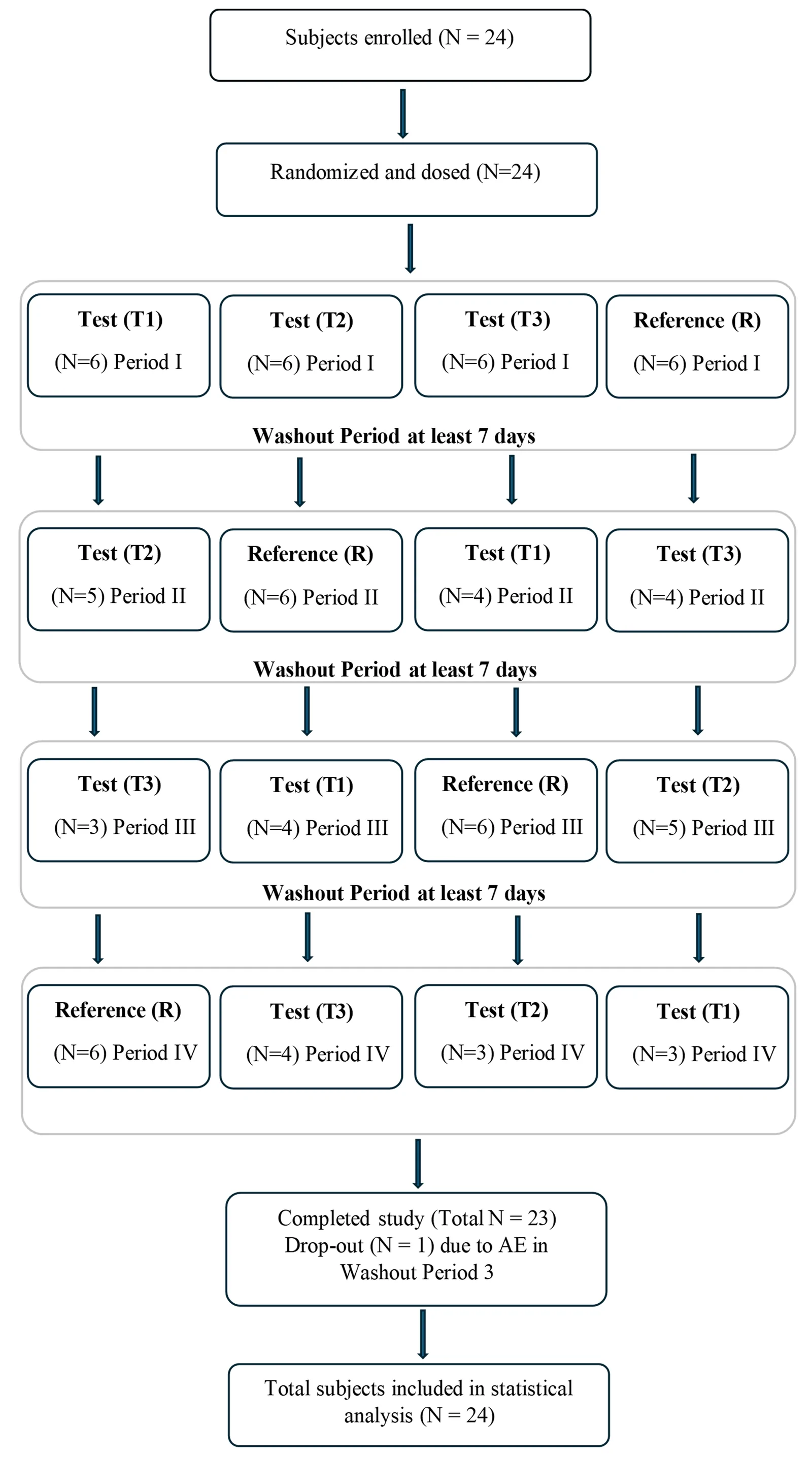
Flowchart of the PK study.

This was a Phase 1, single-dose, open-label, crossover PK study in healthy volunteers under fed conditions, with a 7-day wash-out period between vaginal drug administrations in each treatment period. Twenty-four subjects were randomised into 4 sequences according to the SAS^®^ procedure. The flow diagram summarises the enrolment and participation of subjects (Figure 1).

The rationale for conducting a PK study was to identify the relationship between vaginal drug dose and systemic exposure and to confirm that human in vivo vaginal absorption remains below the systemic levels produced by the 100 mg oral comparator.

The clinical trial was conducted in a single centre in the Czech Republic (Quinta Analytica s.r.o.) from 18 October 2021 to 28 November 2021, in compliance with the EU Directive 2001/20/EC and the ICH GCP. The trial followed ethical principles outlined in the Helsinki Declaration and was approved by the Ethics Committee (IKEM and FTN, M-21-57) and by the national health authority (SUKL, sukls219785/2021). The clinical trial identification number is EudraCT number 2021-003579-32. Ethics approval was obtained solely for the research published in this article.

Subjects provided written informed consent before starting the screening procedures. Prior to dosing, the investigator assessed whether potential study subjects had met the inclusion/exclusion criteria (Table 2 and Table S1).

#### 2.4.2. Treatment

As recommended in the summary of the comparator product characteristics, the drug should be taken orally with meals. Therefore, vaginal and oral tablets were administered under fed conditions. The oral comparator was taken with 240 mL of water. No other food was allowed from at least 10 h before dosing until at least 4 h after dosing. The meals and fluids were standardised and controlled during the study.

The investigational products were administered at the clinical site under the study team’s supervision. Participants were randomly assigned to one of the four treatment sequences. In the PK study, a single dose of vaginal tablet at 5 mg (T1), 25 mg (T2), or 100 mg (T3), or a single oral dose of 100 mg comparator (50 mg dosed as two tablets, C) was administered to participants. A 7-day wash-out period was chosen to allow the complete elimination of the drug before subsequent dosing and to avoid the carry-over effect.

#### 2.4.3. Pharmacokinetic Evaluations

Blood samples were collected over 28 h following administration of investigational medicinal products (IMPs) according to the same sampling schedule. A total of 19 blood samples were collected in each period: pre-dose (−1.00) and at 0.25, 0.50, 0.75, 1.00, 1.50, 2.00, 2.50, 3.00, 3.50, 4.00, 5.00, 6.00, 8.00, 10.00, 12.00, 16.00, 24.00, and 28.00 h post-dose and stored at −70 °C until analysis. All deviations from the scheduled blood sampling time point were recorded and used to adjust the real-time PK evaluation. Plasma drug concentrations (K_2_EDTA) were analysed by Quinta-Analytica s.r.o. using a dual tandem high-performance liquid chromatography and mass spectrometry HPLC-MS/MS method, with ^13^C3-antibacterial agent as the internal standard. Chromatographic separation was achieved on a Kinetex Bifenyl column using a mobile phase of methanol and purified water. The plasma calibration curve ranged from 0.15 to 1000.0 ng/mL, with accuracy and precision meeting EMA acceptance criteria (accuracy within ±15% for all QC levels except LLOQ, where ±20% was accepted; precision as CV% ≤15% or ≤20% for LLOQ). The lower limit of quantification (LLOQ) was 0.15 ng/mL. No significant matrix effects or carry-over were observed. Samples exceeding the calibration range were diluted with blank matrix and reanalysed. Concentration values below LLOQ were set to zero in the PK analysis. The long-term stability of the drug in plasma was confirmed at −20 °C and −70 °C up to 52 days. Stability was confirmed at room temperature, after multiple freeze–thaw cycles, and during autosampler storage.

For PK characterisation, concentrations for each dose were calculated using non-compartmental analysis with the linear trapezoidal method and linear interpolation. Phoenix WinNonlin™ Software version 8.3 (Pharsight, Certara™ Company) and SAS^®^ version 9.4 (SAS Institute Inc.) were used for PK. The actual post-dose sampling intervals (relative to drug IMP administration) were used for all parameter estimations. Standard PK parameters included measures of the extent of absorption using estimates of the area under the plasma concentration–time curve, AUC_(0–t)_, area under the plasma drug concentration versus time curve from time zero to infinity, AUC_(0–∞)_, the maximum drug concentration, C_max,_ and time to reach the maximum drug concentration, T_max_, and terminal half-life, t_1/2_.

#### 2.4.4. Sample Size Determination

As in FIH studies, primary endpoints were principally descriptive. William’s balanced 4-period, 4-treatment, 4-sequence design was applied; a sample size of 6 subjects per sequence (24 subjects per treatment) was selected to adequately characterise the relative bioavailability of the single-dose vaginal product compared with the oral comparator.

#### 2.4.5. Safety and Tolerability

Subject safety was monitored at protocol-specified time points; any adverse events (AEs) were recorded. Assessments comprised local tolerability, gynaecological examinations (cytology, vaginal secretions, tablet residuum), medical history, physical examination, 12-lead ECG, vital signs, haematology, biochemistry, urinalysis, and pregnancy testing.

## 3. Results

### 3.1. IVPT Study

The presented study investigated the safety and absorption profile of an antimicrobial molecule in the form of a bioadhesive vaginal tablet reformulated from an oral comparator. The model drug with broad-spectrum antimicrobial activity, exhibits limited solubility (0.01 mg/mL) when administered orally. Table 3 provides the bioadhesive parameters for vaginal tablets. In contrast to the oral comparator, vaginal tablets contain a non-ionic, hydrophilic polymer. This pharmaceutical ingredient swells moderately on contact with moisture and adheres easily to surfaces. The attained values were adjusted during formulation studies to balance sufficient adhesion to tissue with effective disintegration upon contact with vaginal fluid. The comparator was a conventional 50 mg oral, immediate-release tablet for urinary tract infections.

**Figure 2:**
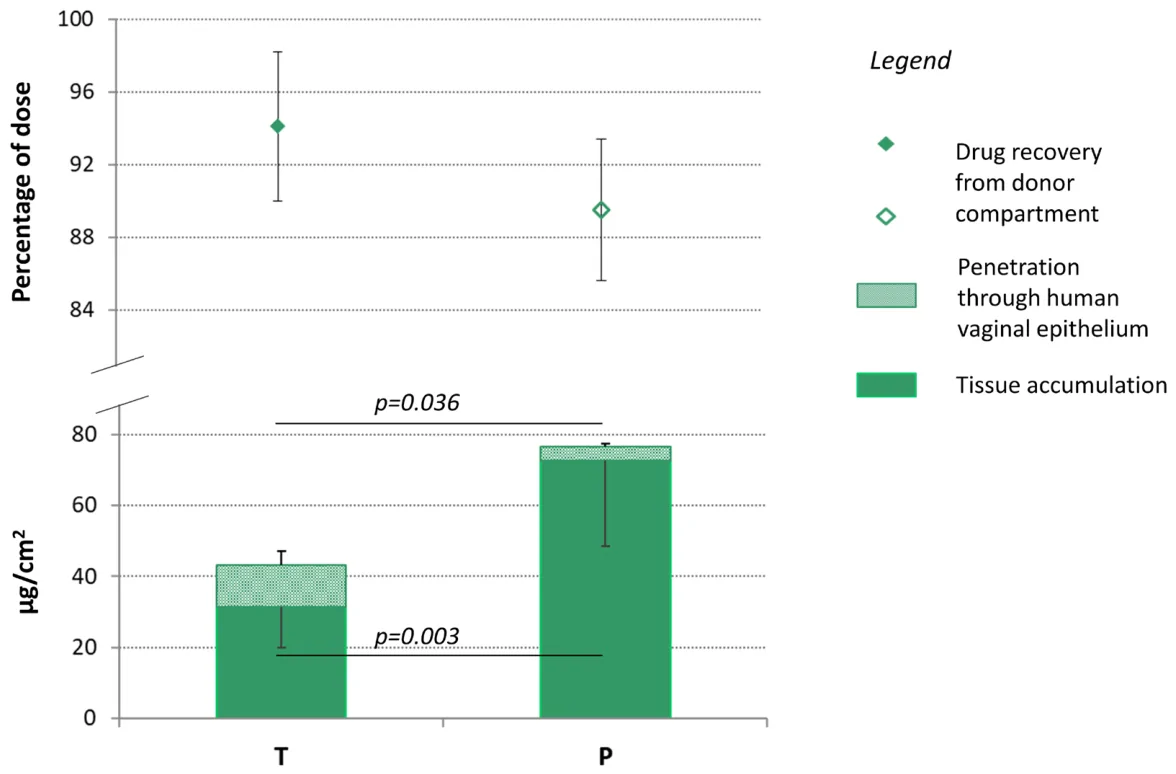
In vitro permeability (penetration and accumulation) of pure drug (P) (mean ± S.D., n = 5) and drug from vaginal tablet (mean ± S.D., n = 10) in human vaginal epithelium (expressed as the amount of drug in acceptor medium per tissue surface area); the amount of drug’s recovery from donor compartment is expressed as a percentage of drug dose placed on the tissue.

The IVPT was used at the product development stage to profile vaginal penetration across excised human vaginal epithelium. Table 4 and Figure 2 present IVPT data for the vaginal tablet (T) vs. the pure antimicrobial drug (P) across excised human vaginal epithelium.

The model drug, which exhibits broad-spectrum antimicrobial activity, has limited solubility (0.01 mg/mL) upon oral administration. An infinite dose of the drug was applied to the tissue surface to achieve maximal penetration. The pH at the tissue surface was outside the physiological range (pH 4.5), simulating an environment that may occur during BV [19]. The molecule (pKa 8.23) was non-ionised at pH 4.5, which should not affect its permeability. In both tests, drug levels in the acceptor fluid were below the limit of quantification at early time points (2, 4, 6, or 8 h). The amount of drug penetrating across excised vaginal tissue into the receptor medium after 24 h of incubation was low, not exceeding 11.6 μg/cm^2^. Drug penetration from the tablet (T) was higher than from the suspension of pure drug (P) (*p* = 0.036) (Figure 2). These differences were most probably due to increased drug solubility (Supplementary Table S2) in the presence of tablet excipients with wetting properties [18]. However, an opposite trend was observed in the retention experiments: pure drug deposition in ectocervical tissue was 2-fold higher than the tablet mass (*p* = 0.003). Overall pure drug permeability (calculated as the sum of the penetrated fraction and drug retention) after 24 h was low, not exceeding 80 μg/cm^2^ (corresponding to 1.58% of the applied dose) for P, and was further reduced to 43.1 μg/cm^2^ (0.9% of the applied dose) for T in the presence of formulation excipients (Figure 2).

### 3.2. PK Study

**Figure 3:**
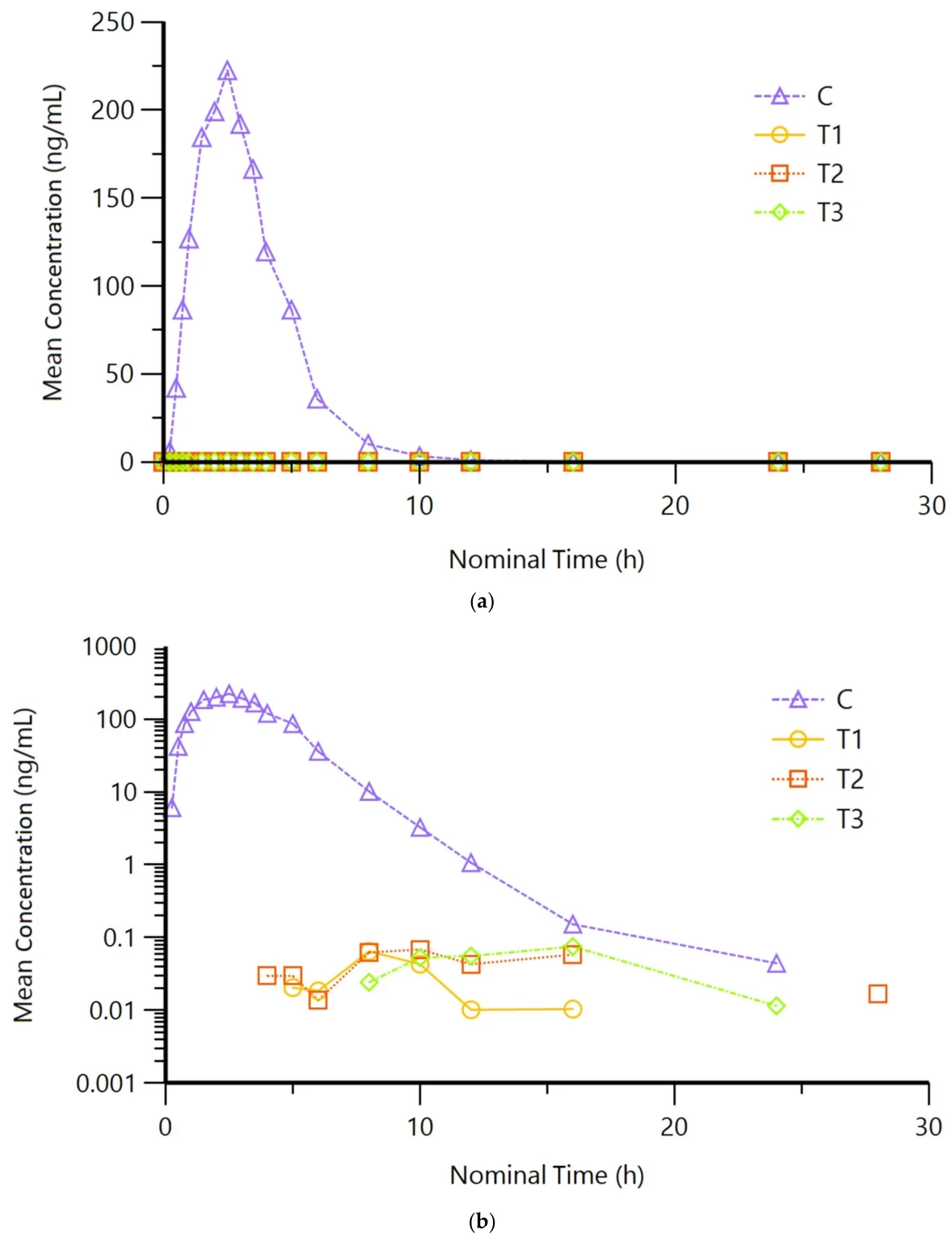
Linear (**a**) or semi-logarithmic scale (**b**) of mean plasma concentration–time profiles for oral comparator (C), bioadhesive vaginal tablet with 5 mg (T1), 25 mg (T2), or 100 mg (T3) of antibacterial agent.

The FIH study used a randomised crossover design to minimise inter-individual variability and bias. Mean plasma concentration–time profiles of the antibacterial drug after single-dose oral and vaginal administration are shown in Figure 3. PK parameters are presented in Table 5.

The number of participants differs across parameters because, for subjects with terminal phase concentrations below the LLOQ, some pharmacokinetic parameters, such as AUC_0–t_ or AUC_0-inf_, could not be mathematically derived because the elimination phase was not quantifiable. In addition, as shown in Figure 1, some participants were not present during all study periods or were excluded from the study.

Analysis revealed limited plasma drug levels after vaginal administration at most time points, with no measurable plasma concentration at the first nine post-dose sampling times. A single vaginal tablet resulted in a low C_max_ (0.084–0.127 ng/mL), irrespective of the applied dose. Mean drug levels were highly variable, with CVs of about 204% (T1), 100% (T2), and 150% (T3). The single maximum plasma concentration of 0.668 ng/mL was observed in the vaginal 5 mg group and was reached after 10 h. Mean T_max_ occurred at 2.3 h (T1), 6.2 h (T2), and 5.0 h (T3). Levels dropped in most participants below LLOQ 24 h post-dose, except for the T3 group, where drug concentration remained quantifiable at 28 h. In six patients (25%), the drug remained below LLOQ throughout the treatment period. Mean AUC_(0–t)_ after vaginal tablets did not exceed 1.56 h·ng/mL (T3) and was significantly lower than for oral 100 mg tablets (824.14 h·ng/mL). The half-time was missing for most subjects, and t½ values (12.37 h and 50.29 h) were assessed only for two individuals in the T2 group. High mean drug concentrations were observed in the oral comparator group; after the 100 mg oral dose, the drug was rapidly absorbed, with C_max_ ranging from 185 ng/mL to 481 ng/mL between 1 and 5 h.

### 3.3. Safety and Tolerability Outcomes

No serious AE leading to discontinuation was reported. Ten subjects experienced a total of three mild and 10 moderate AEs. AEs by body system are listed in Table 6.

No clinically significant changes in laboratory measurements were reasonably attributable to the investigational formulations, except for one case of elevated liver enzyme levels at the outcome examination. The subject fully recovered, as confirmed by the exit examination.

#### Local Tolerability Assessment

No abnormalities were noted at physical or gynaecological examination. Two subjects reported vaginal discomfort: one reported moderate vaginal itching and burning on Day 2 of Study Period 2 after T3, and one reported moderate vaginal burning during Wash-out Period 3 after T3. All events were moderate; 6% of subjects reported itching and 12% reported burning. No clinically significant changes in vaginal microflora were observed by microscopic evaluation.

## 4. Discussion

According to the World Health Organization, topical or oral metronidazole, or vaginal clindamycin, remains the first-line treatment for BV [20]. Although effective, prolonged use may cause severe side effects [21]. Novel, safe, and effective alternatives are needed. The bioadhesive vaginal tablet presented here provides such an option: upon vaginal application, it disintegrates continuously in contact with vaginal fluid, facilitating drug retention in infected mucosal tissue. The investigated antibacterial drug is available as an oral medicinal product registered for genitourinary tract infections. Changing the route of application requires understanding drug absorption and careful PK analysis of the new formulation.

IVPT results showed limited penetration of the model agent across the excised human vaginal epithelium, attributable to its low partition coefficient and poor solubility at acidic pH (<20 µg/mL, Supplementary Table S2). Overall drug absorption (calculated as the sum of penetration fraction and drug retention) from the tablet was 2-fold lower than from pure drug, despite excipients increasing drug solubility by 30% (Supplementary Table S2). This is consistent with Grammen et al., who reported that pharmaceutical excipients increased the solubility of hydrophobic compounds but reduced their permeability [22]. According to Pandey et al., developing vaginal antimicrobials requires a precise balance between mucosal adhesion and intracellular accumulation in infected cells, which act as a drug reservoir between doses [2]. In contrast, Akil et al. observed that increased drug deposition in epithelial cells may facilitate systemic absorption due to proximity to blood circulation [23]. In the present study, tablet excipients reduced drug accumulation in the ectocervical tissue, further hindering absorption.

Only a limited number of studies have investigated IVPT with excised vaginal epithelium so far. For instance, Tsanaktsidou et al. identified log*P*, molecular weight, and water solubility as crucial factors influencing vaginal drug permeability [24]. Szymańska et al. reported that physiological pH changes substantially affect in vitro vaginal absorption of microbicides through human ectocervical tissue [13]. Presented IVPT findings supported PK data by demonstrating a relationship between limited absorption across excised vaginal epithelium and in vivo low plasma concentrations.

The PK analysis directly compared the vaginal formulation across multiple developed doses against the standard 100 mg oral comparator. No significant differences in C_max_/dose or AUC/dose were observed across the three vaginal formulations. The drug exhibits very low aqueous solubility, limiting the amount available for absorption within the small volume of vaginal fluid. According to [25], premenopausal women produce about 2 to 5 mL of mucus-like vaginal discharge every day, and the volume varies, e.g., with the menstrual cycle. In addition, its low lipophilicity (LogP approximately 0.6) restricts passive transcellular diffusion across the vaginal epithelium. Consequently, only a limited fraction of the administered dose reached the systemic circulation, even at the highest dose (100 mg). In contrast, oral administration provides access to substantially larger dissolution volumes and the highly absorptive surface area of the gastrointestinal tract, enabling efficient systemic uptake (Figure 3). Notably, any absorbed fraction is further reduced by the drug’s rapid elimination, with a plasma half-life of approximately 1 h. This comparison confirmed that human in vivo systemic exposure following vaginal administration remains safely below standard treatment levels, thereby establishing a pivotal safety justification prior to initiating dose-finding clinical trials in patients.

Vaginal administration resulted in delayed drug appearance in plasma (T_max_ 0–16 h) compared with the rapid oral peak (1–5 h). According to published sources [26,27], T_max_ and half-life after vaginal application may be delayed, resulting in prolonged plasma levels lasting for hours or even days. The observed PK fluctuations were high, precluding accurate determination of terminal half-life and T_max_. Plasma levels dropped below LLOQ in most participants at 28 h post-dose, with no evidence of drug accumulation.

Notably, the attained plasma PK parameters were markedly lower than those reported for vaginal clindamycin gel, the first-line BV treatment [7]. PK data for clindamycin showed greater systemic absorption, with individual Cmax values of 3.8–236 ng/mL (24 h) and systemic exposure of 62–3822 h·ng/mL. Our mean AUC_(0–t)_ values, not exceeding 1.560 h·ng/mL (100 mg), suggest safer BV therapy with reduced risk of systemic AEs. Clinically, this profile appears beneficial for patients requiring localised antibacterial therapy who may be intolerant of oral antibiotics, such as those with gastrointestinal comorbidities or polypharmacy.

Plasma concentrations consistently below the threshold of clinical concern, together with good local tolerability and safety (AEs were mild to moderate; no participant experienced severe AEs or required discontinuation), support the bioadhesive formulation as a safe alternative to systemic first-line BV treatment.

Limitations include the small sample size of participants receiving a single vaginal dose, which may limit extrapolation to more complex conditions. In healthy volunteers, the vaginal microenvironment may differ from that in patients with active infection, so the observed low systemic absorption may not fully reflect PK in disease.

Inter-subject variability was large, with geometric CV typically >60%, reflecting physiological differences. High PK variability is well documented for hormone vaginal therapies [28]. Te West demonstrated that vaginal cream containing 0.5 mg oestriol produced plasma concentrations ranging from 136 to 1066 pmol/L, due to diverse maturation states of the urogenital epithelium in postmenopausal women [28]. The restricted data density precluded the precise calculation of PK parameters for all subjects, inherently driving high inter-individual variability. Consequently, the estimated summary statistics should be interpreted with caution, as they were calculated from a limited number of quantifiable profiles. Rigorous testing across diverse patient populations is still needed to confirm safety and efficacy.

## 5. Conclusions

This is the first reported study to directly compare human PK and IVPT with excised vaginal tissue, supporting IVPT as a primary tool for predicting vaginal bioavailability and safety. Overall, these observations indicate that the bioadhesive tablet does not favour penetration of the antibacterial drug into the underlying vaginal tissue and should exert only a local effect. A single-dose, randomised, crossover PK study at three different strengths demonstrated that bioadhesive vaginal tablets had good local tolerability and safety. The low active substance absorption following vaginal administration suggests that the drug exerts a localised effect with minimal risk of systemic side effects. Plasma concentrations consistently below the threshold of clinical concern support the bioadhesive formulation as a potential safe alternative to systemic first-line BV treatment. Further studies are still needed to demonstrate adequate local antimicrobial exposure and to identify the dose sufficient for clinical efficacy.

## Figures and Tables

**Table 1:** Testing conditions applied in IVPT studies.

Amount of powdered tablet mass (mg)		75 ± 1 (corresponding to 5 mg of drug)	
Suspending medium		HEC dispersion in SVF (pH 4.5) with composition according to [15]	
Amount of suspending medium (g)		0.5	
Acceptor medium		Sodium chloride	0.9
		Octylphenoxypoly(ethyleneoxy)ethanol (solubilizer)	1.0
		Sodium azide (preservative)	0.005
		Water (pH 6.2)	do 100.0
Flow rate (mL/h)		20	
Sampling	Volume (mL)	1	
	Time intervals (h)	2, 4, 6, 8, 24	
Drug content analysis	HPLC Agilent Technologies 1200, Germany C18-BDS (150 × 4.6 μm, 5 μm); mobile phase ammonium acetate (pH 7.0): ACN 71:29 (1 mL/min); lower limit of quantification 0.08 µg/mL		

**Table 2:** Inclusion criteria.

Inclusion Criteria
Informed Consent Form signed by the subject
Female, non-pregnant, and not breastfeeding
Caucasian, Czech citizenship
Age ≥18 and ≤50 years
Body mass index ≥18.5 and ≤30.0 kg/m^2^
Non-smoker or past smoker
Subjects in good health, as determined by screening medical history, physical examination, gynaecological examination, vital signs assessments, 12-lead electrocardiograms, and clinical laboratory evaluations
Acceptance of the use of highly effective contraceptive measures during the whole study

**Table 3:** Bioadhesion parameters for vaginal tablets T1–T3 and commercial vaginal tablets without bioadhesive excipients.

Parameter	T1	T2	T3	Control
	5 mg	25 mg	100 mg	25 mg
Work of bioadhesion Average [µJ]	18	24	19	7
RSD [%]	42.2	13.0	22.0	43.3

**Table 4:** Quantitative analysis of antibacterial drug from powdered vaginal tablet mass and pure drug in acceptor medium, accumulated in human vaginal epithelium and on the tissue’s surface (drug recovery from donor compartment) after 24 h incubation (mean ± S.D.).

Compartment	Parameter	T	P
Acceptor medium	Concentration of drug per unit tissue area (µg/mL/cm^2^)	0.58 ± 0.35	0.20 ± 0.05
	Total amount of drug per unit tissue area (µg/cm^2^)	11.60 ± 7.04	4.0 ± 1.0
	% drug dose per unit area of the tissue (%/cm^2^) ^1^	0.23 ± 0.17	0.08 ± 0.03
	% of drug dose dissolved in SVF pH 4.5	4.6 ± 2.7	1.80 ± 0.01
Tissue accumulation ^2^	Concentration of drug per unit tissue area (µg/mL/cm^2^)	3.15 ± 1.17	7.25 ± 2.39
	Total amount of drug per unit tissue area (µg/cm^2^)	31.5 ± 11.7	72.5 ± 23.9
	% drug dose per unit area of the tissue (%/cm^2^) ^1^	0.67 ± 0.26	1.50 ± 0.51
Drug recovery ^3^	% drug dose	94.1 ± 4.1	89.8 ± 3.9

^1^ Suspension of pure drug or tablet mass (whose amount corresponded to the drug dose 5 mg) was placed on the tissue surface; concentrations were determined in: ^2^ solvent used for tissue extraction ^3^ in additional control: powdered tablet mass or pure drugs suspended dispersion of HEC in SVF, pH 4.5, incubated for 24 h at 37 °C.

**Table 5:** Pharmacokinetic parameters for plasma drug concentrations after single-dose vaginal tablets (T1–T3) or oral comparator (C) administration.

Parameter	C		T1		T2		T3	
	N	100 mg	N	5 mg	N	25 mg	N	100 mg
AU C_(0–t)_ (h· ng/mL)								
Mean	24	824.147	5	0.891	11	1.054	6	1.560
SD	24	189.600	5	0.510	11	0.965	6	1.023
CV%	24	23.01	5	57.20	11	91.60	6	65.59
Min	24	460.892	5	0.158	11	0.155	6	0.556
Max	24	1166.174	5	1.577	11	3.088	6	3.222
Median	24	850.226	5	0.954	11	0.533	6	1.274
Geometric Mean	24	801.470	5	0.711	11	0.677	6	1.299
AUC_inf_ (h·ng/mL)								
Mean	24	824.722	0	x	2	10.077	0	x
SD	24	189.777	0	x	2	4.861	0	x
CV%	24	23.01	0	x	2	48.24	0	x
Min	24	461.050	0	x	2	6.639	0	x
Max	24	1168.252	0	x	2	13.514	0	x
Median	24	850.597	0	x	2	10.077	0	x
Geometric Mean	24	802.024	0	x	2	9.472	0	x
C_max_ (ng/mL)								
Mean	24	293.306	17	0.084	19	0.127	17	0.099
SD	24	87.024	17	0.172	19	0.127	17	0.149
CV%	24	29.67	17	204.19	19	100.02	17	150.41
Min	24	184.957	17	0.000	19	0.000	17	0.000
Max	24	481.087	17	0.668	19	0.426	17	0.419
Median	24	265.860	17	0.000	19	0.155	17	0.000
Geometric Mean	24	281.754	17	x	19	x	17	x
T_max_ (h)								
Mean	24	2.46	17	2.30	19	6.22	17	5.06
SD	24	1.12	17	3.77	19	6.04	17	7.22
CV%	24	45.64	17	164.40	19	97.12	17	142.64
Min	24	1.00	17	0.00	19	0.00	17	0.00
Max	24	5.00	17	10.02	19	16.02	17	16.03
Median	24	2.50	17	0.00	19	8.00	17	0.00
Geometric Mean	24	2.21	17	x	19	x	17	x

**Table 6:** Listing of adverse events by body system.

Body System	Adverse Event	Serious	Intensity	Relationship	Period	Product
Nervous system	Headache	N	Moderate	Related	1	C
Nervous system	Headache	N	Moderate	Related	1	T3
Nervous system	Headache	N	Moderate	Related	3	C
Nervous system	Headache	N	Moderate	Related	4	T2
Nervous system	Headache	N	Moderate	Related	4	T2
Nervous system	Headache	N	Moderate	Related	4	T2
Nervous system	Headache	N	Moderate	Related	4	T2
Gynaecological disorders	Vaginal discomfort (itching, burning)	N	Moderate	Related	2	T3
Gynaecological disorders	Vaginal discomfort (burning)	N	Moderate	Related	Wash-out 3	T3
Gastrointestinal disorders	Vomiting (6 episodes)	N	Mild	Related	2	T3
Gastrointestinal disorders	Nausea	N	Mild	Related	2	T3
Gastrointestinal disorders	Constipation (bowel obstruction)	N	Mild	Related	Wash-out 3	T3
Gastrointestinal disorders	Elevation of liver enzyme activities	N	Moderate	Related	Wash-out 4	C
Infections and infestations	COVID-19 PCR positivity	N	N.A. ^1^	Not related	Wash-out 3	T2

N—“no”. ^1^ Intensity is N.A.—The subject was absolutely asymptomatic.

## Data Availability

Data are available upon request. To gain access, data requestors will need to sign a data access agreement.

## Supplementary Materials

The following supporting information can be downloaded at https://www.mdpi.com/article/10.3390/pharmaceutics18091183/s1. Table S1: Exclusion criteria; Table S2: Solubility of pure drug (P) in relation to drug from tablet formulation (T) (mean ± S.D., n = 3).

## Abbreviations

The following abbreviations are used in this manuscript:

BV	Bacterial vaginosis
FIH	First-in-human
IVPT	In vitro permeation testing
PK	Pharmacokinetic study
LLOQ	Lower limit of quantification
HPLC-MS/MS	Dual tandem high-performance liquid chromatography and mass spectrometry
