# Supplementary material for: Comparative Analysis of In Vitro Permeation and Human Pharmacokinetic Data for a Bioadhesive Vaginal Tablet as a Potential Clinical Approach for Bacterial Vaginosis
Source: Pharmaceutics. 2026 Sep 19;18(9):1183. doi: 10.3390/pharmaceutics18091183 (PMC13610256; doi:10.3390/pharmaceutics18091183)
Supplement: Supplementary file 1 [file pharmaceutics-18-01183-s001.zip › pharmaceutics-4504350-supplementary.pdf]

SUPPLEMENTARY MATERIAL

Comparative Analysis of In Vitro Permeation and Human Pharmacokinetic Data for a Bioadhesive Vaginal Tablet as a Potential Clinical Approach for Bacterial Vaginosis

Monika Kłosińska [1], Anna Kiryluk [1], Bartłomiej Kubiak [1] and Emilia Szymańska [2,*]

[1] Research and Development Department, Adamed Pharma S.A., Pieńków, Mariana Adamkiewicza 6A, 05-152 Czosnów, Poland
[2] Department of Pharmaceutical Technology, Medical University of Białystok, Mickiewicza 2c, 15-222 Białystok, Poland

* Correspondence: emilia.szymanska@umb.edu.pl; Tel.: +48-(85)7485617

Supplementary Material for Study Design and Participants

Table S1: Exclusion criteria.

**General exclusion criteria**
Gastrointestinal, cardiovascular, renal, or hepatic diseases and/or pathological findings present or in history, which might interfere with the drug pharmacokinetics
Acute or chronic diseases and/or clinical findings that may interfere with the aims of the study or with the drug's safety, tolerability, bioavailability, and/or pharmacokinetics
Clinically significant illness within 28 days before the first dosing, including major surgery
Any significant clinical abnormality, including a positive result of HBsAg and/or HCV and/or HIV test during the screening procedure. (History and presence of increased ALT, AST more than 3x ULN)
Positive urine drug abuse test and/or alcohol breath test at screening and/or at check-in
Positive urine nicotine test at screening
Serious mental disease, difficulty in swallowing solid dosage forms, or acceptability of the intravaginal administration, and/or inability to cooperate with the clinical team
Sitting blood pressure after a minimum of 5 minutes of rest is out of the range of 90-140 mmHg for systolic BP and/or 60-90 mmHg for diastolic BP, and/or heart rate out of the range of 50-100 bpm during the screening procedure
Body temperature out of the range of 35.7-37.0°C at screening
Drug, alcohol (of ≥ 40 g per day pure ethanol), or solvent abuse
Use of organ-toxic drugs or systemic drugs known to substantially alter liver metabolism within 90 days before the first dosing
Use of any prescription medication for a period of 28 days before the first dosing
Any systemic over-the-counter (OTC) drug treatment and/or vitamins and/or herbal treatment and/or food supplements within 14 days before the first dosing

Getting a tattoo, body piercing, or any cosmetic treatment involving skin piercing within 90 days before the screening, unless evaluated by the Investigator as non-significant for inclusion in the study
Donation or loss of at least 500 mL of blood within 90 days or donation of plasma or platelets within 14 days before the first dosing
Haemoglobin below 120 g/L for women
Less than 30 days between the follow-up examination in a previous study and the first dosing in this study
Volunteers who are expected to have difficulty with intravenous catheter insertion or venipuncture
**Study-specific exclusion criteria**
Serum creatinine concentrations out of the acceptable range 49-90 μmol/L, unless evaluated by the Investigator as non-significant for inclusion in the study. Creatinine clearance (GFMDRD-1) is out of the acceptable range
History of polyneuropathy and CNS disease (i.e., frequent attacks of migraine, seizures, etc.)
History of Glucose-6-phosphate dehydrogenase deficiency
History of severe allergy or allergic reactions to the study IMP, its excipients, or related drugs
Topical products applied to the vulva or vagina (e.g., spermicide, hormones, tampons, antibiotics, antifungal, antitrichomonal, corticosteroid, or anti-inflammatory topical products) within 3 days before dosing
Sexual intercourse within 48 h before and after vaginal administration of the IMP
Folate below 12.2 nmol/L at screening
Vitamin B12 below 156 pmol/L at screening
Menstrual bleeding in Period 1 and randomization into a vaginal administration arm in Period 1
Clinically significant vaginal anatomic and functional disorders
Signs/symptoms of vaginal inflammation, vaginal infection, and gynaecological diseases are found in the gynaecological examination
Abnormal Pap smear
Subject with irregular or missing menstrual period
**COVID-19 exclusion criteria**
The subject was vaccinated against COVID-19 less than 14 days before the screening, and/or the subject plans to be vaccinated against COVID-19 during the study
The subject was hospitalised for COVID-19-related reasons
Positive PCR for SARS-CoV-2 within 28 days before screening in subjects with a negative previous COVID-19 history (first positive PCR) or with a COVID-19 history older than 90 days before screening
Positive PCR for SARS-CoV-2 performed within 72 h of the start of hospitalisation

Table S2. Solubility of pure drug in relation to drug from tablet formulation (mean ± S.D., n=3).

**Solvent**		**Concentration (μg/mL)**	
		**Tablet**	**Pure drug**
SVF pH 4.5	2h	21.8±1.7	15.1±4.8
	24h	20.4±1.6	17.7±4.5
Acceptor medium	2h	52.7±2.5	38.3±7.6
	24h	54.8±3.8	33.6±5.3
